# Clinical adoption patterns of 0.35 Tesla MR-guided radiation therapy in Europe and Asia

**DOI:** 10.1186/s13014-022-02114-2

**Published:** 2022-08-22

**Authors:** Berend J. Slotman, Mary Ann Clark, Enis Özyar, Myungsoo Kim, Jun Itami, Agnès Tallet, Jürgen Debus, Raphael Pfeffer, PierCarlo Gentile, Yukihiro Hama, Nicolaus Andratschke, Olivier Riou, Philip Camilleri, Claus Belka, Magali Quivrin, BoKyong Kim, Anders Pedersen, Mette van Overeem Felter, Young Il Kim, Jin Ho Kim, Martin Fuss, Vincenzo Valentini

**Affiliations:** 1 AmsterdamUMC, Loc VUMC, Amsterdam, The Netherlands; 2 ViewRay, Inc., Suite 3000, 1099 18th Street, Denver, CO 80202 USA; 3 Department of Radiation Oncology, School of Medicine, Acibadem MAA University, Istanbul, Turkey; 4 Department of Radiation Oncology, Incheon St. Mary’s Hospital, College of Medicine, The Catholic University of Korea, Seoul, Korea; 5 Radiation Oncology, National Cancer Center Japan, Tokyo, Japan; 6 Radiation Therapy Department, Institut Paoli-Calmettes, Marseille, France; 7 CRCM Inserm UMR1068, Marseille, France; 8 Radiation Oncology, Heidelberg University Hospital, Heidelberg, Germany; 9 Radiation Oncology, Assuta Medical Centers, Tel Aviv, Israel; 10 Radiation Oncology, Ospedale San Pietro Fatebenefratelli di Roma, Rome, Italy; 11 Radiation Oncology, Edogawa Hospital, Tokyo, Japan; 12 Universitätsspital Zürich, University of Zurich, Zurich, Switzerland; 13 Montpellier Cancer Institute (ICM), University Federation of Radiation Oncology of Mediterranean Occitanie, Montpellier University, INSERM U1194 IRCM, 34298 Montpellier, France; 14 Radiation Oncology, GenesisCare-Oxford, Oxford, UK; 15 Radiation Oncology, Klinikum der Universität München, Munich, Germany; 16 Radiation Oncology, Centre Georges-Francois Leclerc, Dijon, France; 17 Department of Radiation Oncology, Sheikh Khalifa Specialty Hospital, Ras Al Khaimah, United Arab Emirates; 18 Department of Oncology, Rigshospitalet, Copenhagen, Denmark; 19 Afdeling for Kræftbehandling, Herlev og Gentofte Hospital, Herlev, Denmark; 20 Radiation Oncology, Chungnam National University Sejong Hospital, Daejeon, Republic of Korea; 21 Department of Radiation Oncology, Seoul National University Hospital, Seoul, Republic of Korea; 22 Radiology, Radiation Oncology and Hematology Dept., Università Cattolica S.Cuore, Fondazione Policlinico Universitario A. Gemelli IRCCS, Rome, Italy

**Keywords:** MRI-guided radiotherapy, MR-IGRT, Stereotactic body radiotherapy, SBRT, Stereotactic ablative radiation therapy, SABR, ART, oART, On-table adaptive radiation therapy, Care patterns

## Abstract

**Background:**

Magnetic resonance-guided radiotherapy (MRgRT) utilization is rapidly expanding, driven by advanced capabilities including better soft tissue imaging, continuous intrafraction target visualization, automatic triggered beam delivery, and the availability of on-table adaptive replanning. Our objective was to describe patterns of 0.35 Tesla (T)-MRgRT utilization in Europe and Asia among early adopters of this novel technology.

**Methods:**

Anonymized administrative data from all 0.35T-MRgRT treatment systems in Europe and Asia were extracted for patients who completed treatment from 2015 to 2020. Detailed treatment information was analyzed for all MR-linear accelerators (linac) and -cobalt systems.

**Results:**

From 2015 through the end of 2020, there were 5796 completed treatment courses delivered in 46,389 individual fractions. 23.5% of fractions were adapted. Ultra-hypofractionated (UHfx) dose schedules (1–5 fractions) were delivered for 63.5% of courses, with 57.8% of UHfx fractions adapted on-table. The most commonly treated tumor types were prostate (23.5%), liver (14.5%), lung (12.3%), pancreas (11.2%), and breast (8.0%), with increasing compound annual growth rates (CAGRs) in numbers of courses from 2015 through 2020 (pancreas: 157.1%; prostate: 120.9%; lung: 136.0%; liver: 134.2%).

**Conclusions:**

This is the first comprehensive study reporting patterns of utilization among early adopters of a 0.35T-MRgRT system in Europe and Asia. Intrafraction MR image-guidance, advanced motion management, and increasing adoption of on-table adaptive RT have accelerated a transition to UHfx regimens. MRgRT has been predominantly used to treat tumors in the upper abdomen, pelvis and lungs, and increasingly with adaptive replanning, which is a radical departure from legacy radiotherapy practices.

## Background

The field of radiotherapy (RT) has seen significant technological advances over the last decades. Of these, image-guided radiotherapy (IGRT) has fundamentally changed the workflow, providing critical information about patient and tumor anatomy on the day of treatment [1]. Conventional IGRT using cone beam computerized tomography (CBCT) or mega-voltage CT (MVCT) has become a standard of care. Both CBCT and MVCT administer incremental ionizing radiation exposure and have notable limitations in soft tissue contrast and image quality that may limit prescribed target dose and adoption of ultra-hypofractionation.

Magnetic resonance imaging (MRI), with superior tissue visualization, has been used in radiation oncology for over a decade. Prior to 2014 and the introduction of the first MRI-guided radiotherapy (MRgRT) system, the use of MRI had been limited mainly to image co-registration during the radiation therapy planning stage. The MRgRT system combines an 0.35 Tesla (T) MRI with a radiation delivery system, initially a tri-Cobalt 60 dose delivery system; since 2017 a linear accelerator. The MR-Cobalt and MR-linac systems allow for superior tumor visualization and radiation beam targeting at the time of treatment [2]. Additionally, software integrated into the system control unit enables daily on-table adaptive planning if the imaging indicates changes in tumor shape or a change in the geometric relationship between target and nearby organs at risk (OAR). Continuous real-time soft tissue tracking during radiation dose delivery with automated beam control (gating) enables reduction of planning target volume (PTV) margins. Combining these technological capabilities holds the promise of better targeting precision, particularly in organ sites where CT-based technologies provide insufficient soft tissue contrast, along with avoidance of OAR.

Among the expected additional benefits of MRgRT compared with existing technologies is the option for dose escalation to the tumor whilst prioritizing dose limits of OAR, thereby, aiming for higher rates of tumor eradication without an increase in toxicity [3]. These ablative radiation doses are typically delivered over a shortened timeframe using ultra-hypofractionated dose schedules (UHfx) such as stereotactic body radiation therapy/stereotactic ablative body radiotherapy (SBRT/SABR).

Despite eight years of clinical use, MRgRT is still generally considered a novel technology, and optimal clinical applications continue to be investigated. However, a growing body of prospective and retrospectively collected data on clinical outcomes for some difficult-to-treat cancers has become available [4–20]. While a number of users has reported on the clinical use of MRgRT, the general pattern of utilization of these systems in Europe and Asia has not been reported [21–23].

Therefore, we conducted this retrospective administrative database study to report utilization patterns of the 0.35T-MRgRT system over time. More specifically, we were interested in assessing the most frequent tumor sites treated, use of UHfx, and adoption of on-table adaptive radiotherapy (oART).

## Methods

The data source for this retrospective, descriptive study was a machine database that maintains a historical record of all treatments delivered on 0.35T-MRgRT systems globally. The database stores information about all fractions beginning with an institution’s first treatment through the date the data were extracted. Data describing number and types of treatments over time and other treatment related information such as the number of fractions, number of oART fractions, tumor site treated were available.

No Protected Health Information (PHI) was collected and none of the extracted data fields allow identification of individual patients. All data comply with applicable law governing data privacy, including but not limited to Health Insurance Portability and Accountability Act of 1996 (“HIPAA”) and the General Data Protection Regulation 2016/679 (“GDPR”).

Records were included for systems installed at institutions in Europe and Asia for completed treatment courses occurring from 2015 through the end of 2020. Since no patient identifiers were collected, commissioning, testing and quality assurance procedures may also have been included in the data collection. In order to purge these procedures, we excluded courses with a planned dose ≥ 100 Gy or planned fractions > 45, as both would be implausible to be clinically prescribed for an actual treatment course.

Detailed fraction-level data was unavailable for some courses treated on Cobalt machines at two institutions. We also excluded treatment courses from a third institution from the analysis of oART fractions because of the unique workflow at the institution that prevented accurate data capture on oART fractions.

A patient could have undergone more than one treatment course in the assessed timeframe, and each would be counted separately. We classified oART treatments if at least one fraction in a treatment course was delivered using an adapted plan. UHfX was defined as five fractions or fewer. Accelerated partial breast irradiation (APBI) was defined as ten fractions and a dose of 38.5 Gy [24, 25].

Analysis was performed using Tableau Desktop 2021 (Seattle, WA, USA) and Excel Office 365 (Microsoft Corporation, Redmond, WA, USA). When detailed treatment data was not available for certain cobalt courses, the total number of missing courses (n = 622) was added back to the overall totals for course-level analyses. Total numbers of oART and UHfx courses were calculated using their respective ratios calculated from the detailed data multiplied by the total, including the cobalt courses. We counted total and average fractions and adapted fractions for all treatment courses. These analyses were stratified by tumor site and UHfx versus non-UHfx fractionation schemes. Growth rates were calculated using compounded annual growth rates (CAGR) methodology [26].

## Results

Between 2015 through 2020, 5796 courses were delivered on 0.35T MRgRT systems in 21 institutions (22 systems) in Europe and Asia, of which 3516 were delivered on an MR-linac and 2280 on Cobalt systems (622 without detailed data) (Table 1). Eighteen of 21 centers (85.7%) had treated for ≥ 1 year, of which 14 delivered > 100 courses/year and 9 > 150 courses/year. The CAGR for total courses was 146.0%, growing from 28 in 2015 to 2522 in 2020.

**Table 1:** 0.35T-MRgRT Utilization in Europe and Asia, 2015–2020

Measure	Total (2015–2020)	2015	2020
# Centers (systems)	21 (22)	1 (1)	21 (22)
# Total Treatment Courses^a^	5796	28	2522
% Ultra-Hypofractionated Treatment Courses (≥ 5 fractions)^b^	63.5%	39.3%	71.6%
% Treatment Courses with ≥ 1 Adaptive Fraction^c^	46.5%	0%	60.0%
# Fractions^b^	46,389	254	19,870
% Adaptive out of Fractions Delivered^c^	23.5%	0%	33.7%
*Treatment sites—distribution*			
Breast	8.0%	57.1%	3.2%
Liver	14.5%	17.9%	14.5%
Lung	12.3%	14.3%	12.1%
Pancreas	11.2%	0%	12.6%
Prostate	23.5%	0%	26.5%
Other^d^	30.4%	10.7%	31.3%

^a^3516 linac courses (all linac courses with detailed data available), 2280 total cobalt courses (1658 cobalt courses with detailed data available). Total n = 5796

^b^For 5174 courses, excluding 622 cobalt courses

^c^For 4977 courses, excluding 622 cobalt courses and 197 courses from a single institution without oART data

^d^24 individual ICD10 diagnosis codes and “undefined” organ sites

**Fig. 1:**
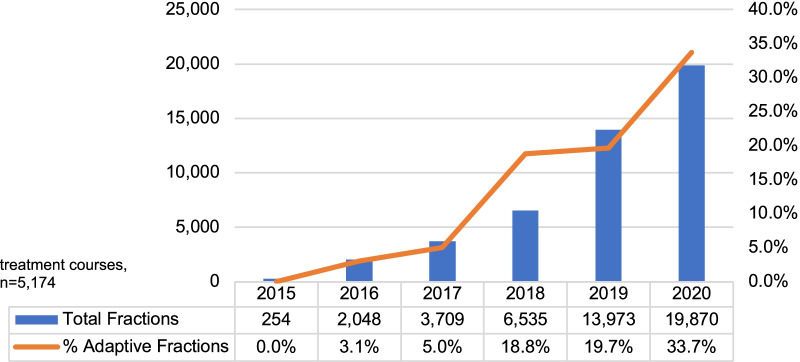
Fractions Delivered on the 0.35T-MRgRT in Europe and Asia—2015–2020

In 5174 courses with detailed data, 46,389 fractions were counted, of which 23.5% were adapted (Fig. 1) The average number of fractions per course was 9.0; the average number of oART fractions was 2.1. Overall growth in the number of fractions delivered from 2015 to 2020 was 139.2% (from 254 fractions to 19,870 fractions).

**Fig. 2:**
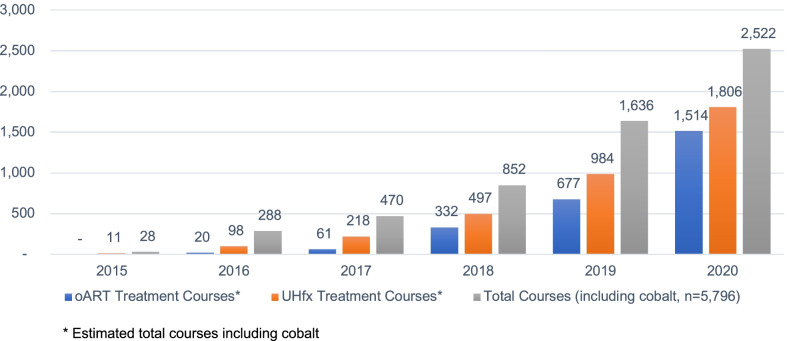
Annual Number of 0.35T-MRgRT Treatment Courses in Europe and Asia—2015–2020

Of these 5174 courses, 63.5% were treated with UHfx and in 4,977 courses with complete oART data 46.5% had at least one fraction adapted (Fig. 2). The growth in UHfx and number of oART courses over the time period was 177.4% and 228.4% respectively. The proportion of courses delivered using UHfx treatment schedules increased from 39.2 to 71.6% and the percent of courses using oART had reached 60.0% by the end of 2020.

The most common organ sites were prostate (23.5%), liver (14.5%), lung (12.3%), pancreas (11.2%), and breast (8.0%). A breakdown by primary versus secondary malignancy treated was not available in this analysis. We grouped all other 24 named organ sites including 15.6% with undefined tumor sites into “other”, which in total represented 30.4% of all treatment courses. The absolute numbers of courses increased over the assessed period with CAGRs of 157.1% (pancreas), 136.0% (lung), 134.2% (liver), and 120.9% (prostate).

**Fig. 3:**
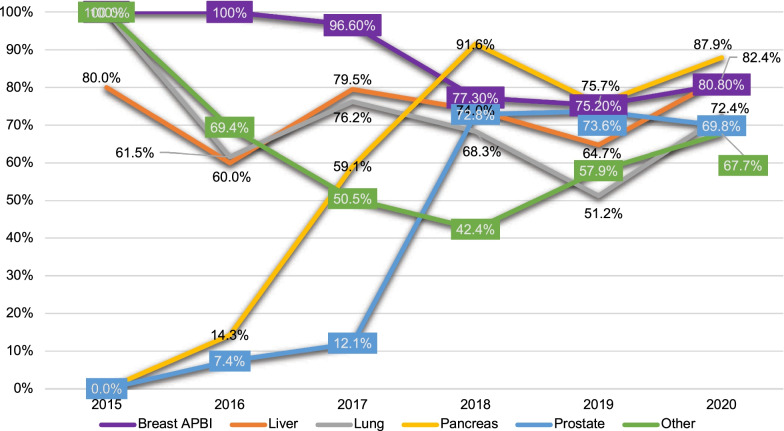
Percent Ultra-hypofractionation and Breast APBI in Europe and Asia—2015–2020

The use of UHfx varied by organ site with overall proportions for pancreas, liver, prostate, and lung of 83.3%, 74.6%, 68.4%, and 65.6%, respectively. Among courses delivered for breast cancer, the proportion treated by APBI was 83.9% (n = 348/415). The rates of UHfx were more variable in the earlier years of clinical use as shown in Fig. 3.

**Fig. 4:**
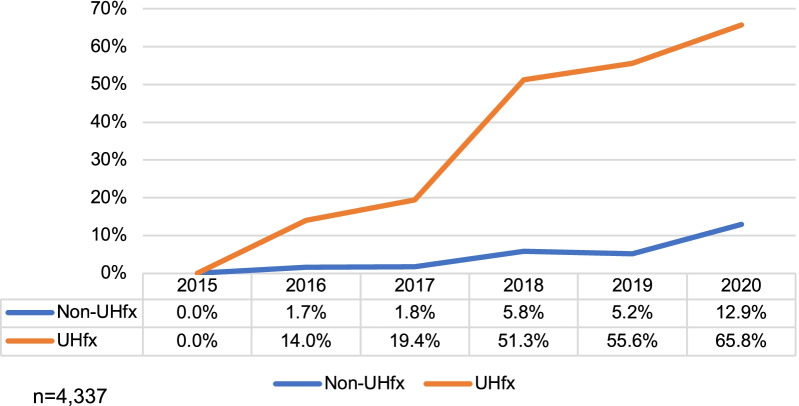
Percent Adaptive Fractions: Ultra-hypofractionation versus Non-Ultra-hypofractionation in Europe and Asia—2015–2020

The proportion of oART fractions in the 4977 courses with available adaptive fraction data increased from 3.1% in 2016 (the first year of oART use) to 33.7% (n = 6490/19,254) by 2020, for a CAGR of 81.9%. In 2020, the proportion of oART fractions was highest in treatment courses for pancreas (59.1%), lung (36.4%), liver (33.2%), and prostate (33.3%). The proportion of fractions adapted in UHFx courses was 57.8%, with 65.8% of fractions (n = 4980/7569) adapted in 2020 (Fig. 4). In non-UHfx courses, the percentage of oART fractions was 7.6% and 12.9% in 2020. The average number of fractions was 16.6 and 1.3 (total and oART) in non-UHfx courses and 4.6 and 2.7 in UHfx courses, respectively. Variation in the average fractions over time are found in Table 2.

**Table 2:** Average number of fractions delivered by 0.35T-MRgRT in Europe and Asia, 2015–2020

Fractions per course	Treatment type	2015	2016	2017	2018	2019	2020
Mean all fractions	Non-UHfx	11.6	15.6	17.5	16.6	15.9	17.2
	UHfx	5.2	3.9	4.6	4.8	4.6	4.6
Mean oART fractions	Non-UHfx	0	0.3	0.3	1.0	0.8	2.2
	UHfx	0	0.6	0.9	2.4	2.5	3.0

## Discussion

With almost 6000 treatment courses analyzed, this is the largest multi-institutional study to date to report comprehensively on patterns of clinical utilization among adopters of the 0.35T-MRgRT system in Europe and Asia and represents roughly 50% of all patients treated on MRIdian systems (ViewRay Inc, Oakwood Village, OH) worldwide. A similar report detailing use of the technology is under preparation for United States (US) sites.

We observed that shortened treatment courses were increasingly adopted, with nearly 72% of courses delivered using UHfx in 2020. Some organ sites such as pancreas had an even higher proportion of UHfx at nearly 88%. For non-UHfx courses, the average number of fractions (17.2 in 2020) was still substantially lower than standard courses of RT using other technologies which typically range from 20 to 45 fractions [27–30].

UHfx may be delivered using a conventional linac equipped with CBCT or MVCT or other dose delivery systems, but for many organ sites, requires insertion of radio-opaque fiducial markers and usually does not allow intrafraction monitoring of the target’s position or the position of nearby OAR. Even though UHfx has been in use for many years, it has had relatively slow adoption because of concerns about toxicity, lack of long-term data, limited availability of technology in certain geographic areas, and financial disincentives [31]. A 2015 survey of use of SBRT in Europe found that all 30 responding centers offered SBRT for early-stage, non-small cell lung cancer (NSCLC), while only one in three sites offered SBRT for primary liver tumors, 13.3% for pancreas, and 13.3% for prostate also [32]. SBRT for oligometastases, such as lung and liver metastasis was also offered by most centers. However, this survey of centers did not provide insight into the actual utilization of SBRT for patients’ treatments across tumor sites.

In 2017, Holmes et al. reported on the frequency of use of SBRT for stage IA NSCLC based on an analysis of the US National Cancer Data Base [33]. By 2013, SBRT was offered to 16.3% of all stage IA NSCLC, while conventionally fractionated RT was offered to 10.8%. It is interesting to note that SBRT use had increased by over 240% from 6.7% in 2008, while conventional RT had seen moderate decline. While MRI is not frequently used in the diagnostic workup for lung cancer, the significant number of lung cancer patients undergoing UHfx treatment on the 0.35T MRIdian system highlights the ability to avoid susceptibility artifacts on a low-field MRI that may obscure the visualization of small parenchymal lung lesions on higher field-strength MR-linac systems [34].

Several studies have examined the adoption of SBRT in prostate cancer, mostly from a US perspective. Although there has been an upward trend in adoption over the past ten years [35–37], the percentage of SBRT use in the US was only 7.2% in 2015 [38]. In sharp contrast, the present analysis shows nearly 70% of prostate patients treated on 0.35T-MRgRT systems received their treatment by SBRT in 2020. While the above data is not directly comparable, centers report that they prefer MRgRT for prostate patients when SBRT dose schedules are considered. Also, MRIdian centers frequently report that they establish SBRT prostate programs with the clinical go-live of the MRIdian system because of their increased confidence to deliver potentially harmful radiation doses safely.

SBRT in pancreatic cancer is not yet widely adopted with only 2.3% of patients treated by UHfx [39]. Notably, by 2020, 88% of treatment courses delivered for pancreatic cancer on the 0.35T MRgRT system were treated with UHfx. While the total number of pancreas cancer patients undergoing RT has dwindled over the last decade, based on a number of clinical trials documenting a lack of clinical benefit when delivered in conventional fractionation and low biologically effective doses, recent retrospective data suggest that SBRT delivered at ablative dose levels on the 0.35T MRIdian system may result in favorable survival rates at two years while avoiding feared radiation-related higher-grade toxicities [8, 11, 16] With enrollment in the to-date largest prospective clinical trial assessing safety and efficacy of MR-guided SBRT for locally advanced pancreatic cancer completed, outcomes data on early trial endpoints will become available as early as 2022 to further guide decision making and stratification of patients to undergo optimal treatment [NCT 03621644].

In this large series, MRgRT using a 0.35T imaging system has predominantly been employed to treat tumors in the upper abdomen, pelvis and thorax, with prostate, liver, lung and pancreas the most frequently treated. Earlier reports on MRgRT clinical practice patterns had come from single institutions. In 2018, Henke et al. reported on 666 treatment courses delivered on the 0.35T tri-Cobalt MRgRT system over the first 4.5 years of clinical use (2014–2018) [21]. The most common disease sites were breast (31.4%), pancreas (15.2%), liver (13.1%), lung (10.1%), and prostate (5.3%), with 39.9% of courses delivered by SBRT and 13.3% of all fractions adapted. Patients were stratified to undergo MRgRT for improved soft-tissue visualization (14%), continuous real time imaging and gating for respiration motion management (57.5%) and on-table or offline adaptive radiation therapy (28.5%), respectively. Notably MRI-based image-guidance was seen as a benefit for a rather small subset of patients, but real-time soft tissue tracking and gating and the ability to adapt a plan when needed were the most common rationales to treat a patient by MRgRT. Data summarizing early use of a 0.35T MR-linac in Turkey reported on 72 patients treated for 84 malignant lesions [22]. Most common treatment sites included abdomen (43%), pelvis (34%), and lung (21%), with 90.2% of patients treated by SBRT and 93.2% undergoing oART. A second paper from the same group reported on 154 patients treated by adaptive treatment courses to 166 treatment sites [23]. The authors report using adaptive replanning because of lack of target volume dose coverage (56.8%), OAR dose violations (10.7%), or both (24.6%). Similar data could not be extracted in the present analysis.

The availability of MR-linac systems is no longer limited to 0.35T-based systems with a 1.5T-based MRgRT system now also commercially available. In 2021, the MOMENTUM study reported patterns of care for the 1.5T MR-linac based on 516 patients with available treatment data [40]. It is noteworthy that this report summarizes data for system use worldwide and not limited to Europe and Asia as in the present report. Although the types of tumor sites treated show similarities between the systems, the distribution in numbers of patients treated by organ site was different with 40% (vs. 23.5% at 0.35T) prostate, 5% (vs. 14.5%) liver, 4% (vs. 11.2%) pancreas and 1% (vs. 12.3%) lung tumors treated. Aside from differences in use of the systems for lung, liver and pancreas, use of the 1.5T-based systems for brain and head and neck (H&N) stands out at 14% of total. Neither brain nor H&N cancer reach even 1% of use in the present data.

The use of adaptive radiation therapy on the 1.5T MR-linac is reported as “adapt to shape”, most comparable to oART as in the present report, and “adapt to position”, a compensation for a non-moving treatment table, more comparable to classic image-guided radiation therapy. Adapt to shape was used in 64% (n = 328) of treatment courses, implying that this capability is one of the defining features to stratify patients to any of the two available MR-linac systems [40]. When comparing frequency of use of oART between the systems, again similarities and differences emerge. While the frequency of oART use for pancreas cancer is comparably high, with 59.1% (0.35T) and 76% (1.5T), and reasonably comparable for liver with 33.2% versus 17% of use, respectively, the frequency of use of oART in prostate cancer shows surprising differences. While one in three courses for prostate cancer contain adapted fractions on the 0.35T MR-linac system, only 5% of courses contained adapted fractions on the 1.5T MR-linac system. This difference in the frequency of use of oART is particularly notable as the median number of fractions for prostate cancer was 5 in both the present series and the Momentum consortium report, thus likely representing SBRT to similar dose levels. It will be interesting to see if use data homogenizes more with increasing use of the two systems.

Despite the large number of treatment courses presented here, the current analysis has limitations. First, the source of the data was the 0.35T-MRgRT systems and was originally  collected as part of the clinical workflow process and not for prospective research purposes. Therefore, the analysis was limited to the availability and detail of data collected for that purpose. This is most notable in the large proportion of courses administered for “other” malignancies. While most of these probably represent oligometastases, we cannot with certainty state that this is the case. The treatments here are reported by organ site, and specifically for lung and liver, we were not able to differentiate between treatments for primary tumors versus treatment for metastatic disease. Also, some data is incomplete with respect to fraction detail. As such, this may not have allowed to fully capture the adoption rate of oART and UHfx on the systems over time, with numbers presented likely showing an underrepresentation of both to some degree, especially for early years (2016–2018). Lastly, outcomes data linked to this dataset was unavailable. However, peer-reviewed evidence on clinical outcomes for 0.35T-MRgRT with or without oART in both prospective and retrospective series continues to grow [4–20]. Ongoing and completed prospective trials awaiting maturation of outcomes data will provide additional insights in the coming years.

## Conclusions

In conclusion, an accelerated transition to ultra-hypofractionated regimens and on-table adapted radiation therapy on 0.35T-MRgRT systems in Europe and Asia was observed. It has enabled a significant proportion of treatments to be delivered in shortened treatment courses and to ablative dose levels and has facilitated treatments of highly complex cases such as liver and pancreatic tumors. Along with the use data presented here, a series of clinical papers show promise of better patient outcomes, which may lower healthcare costs, ease access to radiation therapy services and improve patient convenience.

## Data Availability

Original machine data for this study is not available due to the need to respect institution confidentiality.

## Abbreviations

APBI: Accelerated partial breast irradiation
ART: Adaptive radiotherapy
CAGR: Compound annual growth rate
CBCT: Cone beam computerized tomography
GDPR: General Data Protection Regulation
Gy: Gray
H&N: Head and neck
HIPAA: Health Insurance Portability and Accountability Act
IGRT: Image-guided radiation therapy
linac: Linear accelerator
MRgRT: Magnetic resonance-guided radiotherapy
MRI: Magnetic resonance imaging
MVCT: Mega-voltage CT
NSCLC: Non-small cell lung cancer
oART: On-table adaptive radiation therapy
OAR: Organs at risk
PTV: Planning target volume
PHI: Protected health information
RT: Radiation therapy
SABR: Stereotactic ablative radiation therapy
SBRT: Stereotactic body radiotherapy
T: Tesla
UHfx: Ultra-hypofractionated
